# Integrated Analysis of Ferroptosis-Related Biomarker Signatures to Improve the Diagnosis and Prognosis Prediction of Ovarian Cancer

**DOI:** 10.3389/fcell.2021.807862

**Published:** 2022-01-05

**Authors:** Huan Wang, Qi Cheng, Kaikai Chang, Lingjie Bao, Xiaofang Yi

**Affiliations:** ^1^ Department of Gynecology, Hospital of Obstetrics and Gynecology, Fudan University, Shanghai, China; ^2^ Department of Gynecology, Qingpu Branch of Zhongshan Hospital Affiliated to Fudan University, Shanghai, China; ^3^ Shanghai Key Laboratory of Female Reproductive Endocrine Related Diseases, Shanghai, China

**Keywords:** ovarian cancer, ferroptosis-related genes, diagnosis, prognosis, immunotherapy, chemotherapy

## Abstract

Ovarian cancer remains the most lethal gynecological malignancy. Ferroptosis, a specialized form of iron-dependent, nonapoptotic cell death, plays a crucial role in various cancers. However, the contribution of ferroptosis to ovarian cancer is poorly understood. Here, we characterized the diagnostic, prognostic, and therapeutic value of ferroptosis-related genes in ovarian cancer by analyzing transcriptomic data from The Cancer Genome Atlas and Gene Expression Omnibus databases. A reliable 10-gene ferroptosis signature (HIC1, ACSF2, MUC1, etc.) for the diagnosis of ovarian cancer was identified. Notably, we constructed and validated a novel prognostic signature including three FRGs: HIC1, LPCAT3, and DUOX1. We also further developed a risk score model based on these three genes which divided ovarian cancer patients into two risk groups. Functional analysis revealed that immune response and immune-related pathways were enriched in the high-risk group. Meanwhile, the tumor microenvironment was distinct between the two groups, with more M2 Macrophage infiltration and higher expression of key immune checkpoint molecules in the high-risk group than in the other group. Low-risk patients exhibited more favorable immunotherapy and chemotherapy responses. We conclude that crosstalk between ferroptosis and immunity may contribute to the worse prognosis of patients in the high-risk group. In particular, HIC1 showed both diagnostic and prognostic value in ovarian cancer. *In vitro* experiments demonstrated that inhibition of HIC1 improved drug sensitivity of chemotherapy and immunotherapy agents by inducing ferroptosis. Our findings provide new insights into the potential role of FRGs in the early detection, prognostic prediction, and individualized treatment decision-making for ovarian cancer patients.

## Introduction

According to the statistics of the American Cancer Society in 2020, over 21,000 women were diagnosed with ovarian cancer, of which nearly 14,000 women died from this disease (Siegel et al., 2020). Despite the application of two newly developed molecularly targeted drugs, PARP inhibitors (PARPi) and antiangiogenic agents, ovarian cancer remains the most lethal gynecologic malignancy (Ke et al., 2020). Due to the late diagnosis and lack of long-term effective treatments, the 5-years survival rate of ovarian cancer is only 47.8% (Zhang et al., 2016; Block et al., 2020). It is predicted that the mortality rate of patients with ovarian cancer will rise significantly by 2040 (Bray et al., 2018). Ovarian cancer with distinct clinicopathological and molecular genetic features exhibits a high degree of heterogeneous (Labidi-Galy et al., 2017). Early detection of ovarian cancer has great promise to improve clinical outcomes, however, there are few specific early symptoms or sensitive biomarkers for the screening and prognosis prediction of ovarian cancer by far. Thus, identification of novel potential diagnostic and prognostic biomarkers, as well as innovative therapeutic targets of ovarian cancer is urgently needed to improve patient outcomes.

Ferroptosis is a newly recognized form of non-apoptotic cell death driven by the iron-dependent catastrophic accumulation of lipid reactive oxygen species (ROS), which is morphologically, biochemically, and genetically distinct from traditional cell death such as apoptosis, necrosis, and autophagy (Dixon et al., 2012). Various signaling pathways have been confirmed participating in ferroptosis cell death including the Hippo pathway (Yang et al., 2019), MAPK pathway, and P53 pathway (Jiang et al., 2015; Hattori et al., 2017). In parallel, an increasing number of ferroptosis-related genes (FRGs) have been identified besides two classical ferroptosis-regulated genes SLC7A11 and GPX4, such as Nrf2, ATF4, FSP1 (Chen et al., 2017; Xie et al., 2017; Bersuker et al., 2019; Dodson et al., 2019), etc. With the deepening of research on ferroptosis, emerging studies revealed that ferroptosis is implicated in a broad spectrum of human diseases including cancer, degenerative diseases, carcinogenesis, stroke, intracerebral hemorrhage, traumatic, brain injury, ischemia-reperfusion injury, and kidney degeneration (Stockwell et al., 2017). Targeting ferroptosis-related genes (FRGs) to trigger ferroptosis cell death as novel therapeutic approaches for cancer diagnosis and treatment has attracted considerable attention (Liang et al., 2019). A previous study showed that ovarian cancer stem cells were sensitive to the ferroptosis inducer elastin *in vitro* and *in vivo* (Basuli et al., 2017). Besides, researchers found that induce ferroptotic cell death in ovarian cancer cells *via* activation of the TAZ-ANGPTL4-NOX2 axis provided a promising therapeutic implication (Yang et al., 2020).

Immunotherapy has shown great clinical value in the treatment of ovarian cancer. The most recent study demonstrated that ferroptosis was immunogenic, and ferroptosis cancer cells in the early death stages could induce an adaptive immune response to perform antitumor effects (Efimova et al., 2020). One of the most typical hallmarks of cancer is resistance to apoptosis (Hanahan and Weinberg, 2011), thus induction of new type cell death methods and individualized immunotherapy are becoming hotspots in tumor therapy. To date, the significance of FRGs in diagnosis, prognosis, immunotherapy, and chemotherapy of ovarian cancer has been rarely studied.

In the present study, we comprehensively analyzed the role of FRGs in ovarian cancer and highlights their diagnostic, prognostic, therapeutic potential for ovarian cancer for the first time. By using public databases, we screened consistently dysregulated FRGs as reliable diagnostic and prognostic biomarkers of ovarian cancer. Among these, HIC1 was found to be of both diagnostic and prognostic value. Furthermore, a risk prediction model related to FRGs was constructed. Functional enrichment analysis surprisingly indicated that immune-related processes and pathways were enriched. More importantly, we analyzed the tumor microenvironment (TME), assessed immunotherapy and chemotherapy response between high-risk and low-risk groups. Finally, we performed experiments *in vitro* to explore the association between HIC1 and treatment response in ovarian cancer. Our work provided potential chemotherapeutic and immunotherapeutic strategies due to ferroptosis for ovarian cancer treatment.

## Materials and Methods

### Data Source

The mRNA expression profiles data for 57 ovarian cancer patients and 12 normal samples in the GSE66957 dataset (https://www.ncbi.nlm.nih.gov/geo/query/acc.cgi?acc=GSE66957) were downloaded from the GEO database (https://www.ncbi.nlm.nih.gov/gds). The transcriptome data and corresponding clinical features of 379 ovarian cancer patients were obtained from the TCGA database. After removing the samples with incomplete clinical information, 374 ovarian cancer patients were randomly assigned to the training set (*n* = 261) and validation set (*n* = 113) at the ratio of 7:3 for the subsequent analyses. The data obtained from TCGA and GEO datasets were both publicly available. Then, 288 FRGs were downloaded from the FerrDb database (http://www.zhounan.org/ferrdb/). After removing the overlapped genes, the remaining 259 FRGs were used for our further analyses.

### Analysis of Differentially Expressed Ferroptosis-Related Genes

All gene expression profiles of the present study were normalized and determined by using the scale method in the “limma” R package. Differential gene expression analysis was performed by comparing tumor tissues to normal tissues using the “limma” package of R in the GSE66957 dataset. Genes that meet the threshold of *P*-value < 0.05 and |log_2_ FC| > 1 were considered as DEGs. Subsequently, we intersected the DEGs acquired from the GSE66957 dataset with FRGs to get the DE-FRGs**(** Supplementary Table S1 **)**.

### Construction of the Least Absolute Shrinkage and Selection Operator Model and Receiver Operating Characteristic Curve Analysis in the GSE66957 Dataset

The least absolute shrinkage and selection operator (LASSO) regression arithmetic that uses regularization to improve prediction accuracy was used to identify the feature DE-FRGs from the 60 DE-FRGs (Friedman et al., 2010) and conducted by “glmnet” of the R package. To distinguish patients with ovarian cancer from controls, the expression profile of gene signature was extracted to construct the LASSO model by “glmnet” of the R package. A model index for each sample of the GEO database was further constructed using the regression coefficients obtained from the LASSO analysis. To evaluate the diagnostic ability of the LASSO model constructed by gene signature to identify ovarian cancer, we performed ROC curve analysis in the GSE66957 dataset using the pROC package.

### Construction and Validation of a Prognostic Ferroptosis-Related Gene Signature in the TCGA Database

The FRGs associated with overall survival (OS) in ovarian cancer were determined using univariate Cox regression analysis. Then, a 3-gene signature was constructed with multivariate Cox regression analysis by the R package “glmnet.” The risk score calculating formula we used was:

ExpGene1∗Coef1+ExpGene2∗Coef2+ExpGene3∗Coef3…

where Coef represents the regression coefficients of genes and Exp denotes the normalized expression values of each signature gene. The corresponding patients in the training and validation sets of the TCGA database were classified into high and low-risk groups based on the median values obtained from the risk scores calculated in the above equation. To assess the prognostic prediction reliability of a risk scoring system constructed from the 3-gene signature, we performed a time-dependent ROC curve using the “survivalROC” in the R package. The OS between two risk groups was analyzed by Kaplan-Meier analysis (K-M). Univariate and multivariate Cox regression analyses were carried out to determine independent prognostic predictors of ovarian cancer patients. The nomogram and decision curve analysis (DCA) were conducted to demonstrate the effectiveness of this risk model.

### Gene Set Enrichment Analysis and Evaluation of Immune Microenvironment

The DEGs with *p* < 0.05 and |log_2_ FC| >1 between the high-risk group and low-risk group were utilized to conduct GSEA based on the cluster Profiler of R package. We performed GSEA enrichment analysis by ranking all differential genes according to their differential multiplicity between high and low risk groups. The threshold for enrichment results was set to |Normalized Enrichment score (NES)| > 1 and adjusted p-value < 0.05, and the enrichment results were sorted by adjusted p-value, with immune-related processes and pathways being the top enriched pathways. Then, *P*-values were adjusted by using the BH methods. Immune and stromal scores were calculated based on the ESTIMATE algorithm and were compared between the two risk groups (Yoshihara et al., 2013). With the CIBERSORT algorithm, based on the LM22 gene signature file, we analyzed the infiltration differences of 22 immune cell types between two risk groups of ovarian cancer patients (Newman et al., 2019). The threshold of adjusted *p <* 0.05 was considered significant.

### Impact of Risk Scoring System on Ovarian Cancer Patients Receiving Immunotherapy and Chemotherapy

The expression patterns of the key immune checkpoint molecules in the high- and low-risk groups were further analyzed (Jia et al., 2021). Besides, the Tumor Immune Dysfunction and Exclusion (TIDE, http://tide.dfci.harvard.edu/) algorithm and SubMap were applied to predict the reaction to the immune checkpoint blockade (CTLA-4, PD-1, and PD-L1) in patients with high- and low-risk groups. Furthermore, the pRRophetic algorithm was utilized to monitor the response of chemotherapy to the ovarian cancer patients of TCGA database (Geeleher et al., 2014). To identify effective drugs for the treatment of ovarian cancer, the half-maximal inhibitory concentration (IC50) values for each TCGA-ovarian cancer sample were obtained from the Genomics of Drug Sensitivity in Cancer (GDSC, www.cancerrxgene.org/) database.

### Cell Culture

IOSE80 and human ovarian cancer cell lines OVCAR5, A2780, SKOV3, HEY, ES-2 cells were purchased from Cell Bank of China Science Academy (Shanghai, China). IOSE80 and OVCAR5 were maintained in 1640 medium (Hyclone, United States). A2780, SKOV3, HEY, ES-2 A2780, SKOV3, HEY, and ES-2 were maintained in DMEM medium (Hyclone, United States). The medium was supplemented with 10% FBS (Gibco, United States) and 1% penicillin/streptomycin. Cells were incubated at 37°C in a humidified incubator containing 5% CO_2_. Cell lines were authenticated using short tandem repeat DNA profiling.

### Western Blotting

Cells were seeded in 6-well plates at a density of 1.5×105 cells per well and cultured for 24 h. Cells with or without transfection were collected on ice in RIPA (P0013B, Beyotime Biotechnology, Shanghai, China) and protease Inhibitor Cocktail (HY-K0010, MCE, China). Then, samples were incubated for 30 min at 4°C with shaking and centrifuged at 12,000 g for 30 min at 4°C, and the supernatants were collected. Protein concentrations were measured with a BCA protein assay kit (P0012, Beyotime Biotechnology, Shanghai, China). Proteins were separated using 10% sodium dodecyl sulfate-polyacrylamide gel electrophoresis (P0012A, Beyotime Institute of Biotechnology, Shanghai, China) and transferred to PVDF membrane (Millipore, Billerica, MA, United States) before blocking with 5% non-fat milk in TBST (Tris-buffered Saline, 0.1% Tween-20). Membranes were incubated overnight at 4 °C in a solution containing the following primary antibodies: HIC1 (24949-1-AP, ProteinTech, Wuhan, China, 1: 1000 dilution), actin (66009-1-Ig, ProteinTech, Wuhan, China, 1:20,000 dilution). Subsequently, blots were incubated with an HRP-conjugated secondary antibody (Jackson Immunoresearch, West Grove, PA) with dilution 1:2000 for 1 h at room temperature. Protein expression was detected with ECL reagents (G2020, Servicebio, Wuhan, China) using the enhanced chemiluminescence (ECL) detection system, and quantified by densitometry using ImageJ.

### Cell Counting Kit-8 Assay

Cell viability was detected by Cell Counting Kit-8 (CK04, Dojindo, Kumamoto, Japan). Cells seeded at 5000 cells/well density 96-well plates, 24 h later cells were treated with cisplatin, paclitaxel, or BMS-1 (HY-17394, HY-B0015, HY-19991, MCE, China) for 24 h. Treatment drug concentrations were as follows: cisplatin at doses of 0, 5, 10, 20, 40, 80 µM, paclitaxel at doses of 0, 5, 10, 20, 40, 80 nM, BMS-1 at doses of 0, 2.5, 5, 10, 20, 40 µM. For Fer-1(SML0583, Sigma-Aldrich, United States) or DFO(D9533, Sigma-Aldrich, United States) treament after transfection of HIC1 siRNAs, after transfected with siRNAs for 48 h, cells were treated 2uM Fer-1 or 20 uM DFO for 24 h. Then, 10 µL CCK-8 was added to each well of the 96-well plate and co-incubated with cells for 1 h. Then, the OD values at 450 nm were detected.

### MDA, GSH, and GSSG Measurement

MDA levels were quantified by using a lipid peroxidation MDA assay kit (A003-2-2, Nanjing Jiancheng Bioengineering Institute, China). according to the manufacturer’s instructions. The intracellular concentration of total GSH and oxidized glutathione (GSSG) was measured using a GSH and GSSG Assay Kit (S0053, Beyotime Biotechnology, China) according to the manufacturer’s protocol. Then, use the following formula to calculated reduced GSH concentration: [GSH] = [total GSH]−2*[GSSG].

### Transfection of siRNA and Plasmid

Cells were seeded at a density of 1.5 × 10^5^/well in 6-well plates, and cultured to 70–80% confluent for transfection of control siRNA, HIC1 siRNA, control plasmid (pcDNA3.1), and HIC1 overexpressing plasmid. All siRNAs were purchased from Guangzhou RiboBio (Guangzhou, China). Plasmids were synthesized by General Biosystems (Anhui, China). Lipofectamine 3000 (ThermoFisher, L3000008) was used for siRNA and plasmids transfection according to the manufacturer’s protocol. The knockdown and overexpression efficiency were determined by Western Blotting 48 h post-transfection.

### Statistical Analysis

All bioinformatic statistical analyses of our present study were performed in R (version 3.6.1) software. Differences between the high- and low-risk groups were compared with the Wilcoxon test. All experiments were independently repeated more than three times. Statistical analyses of *in vitro* experiments were performed using GraphPad Prism 8 software and statistical significance was determined by Student’s t-test. All reported *P* values had been passed a two-tailed test and *p* < 0.05 was considered as statistically significant (∗, *p* < 0.05; ∗∗, *p* < 0.01; ∗∗∗, *p* < 0.001).

## Results

### Diagnostic Prediction Performance of FRGs in Ovarian Cancer Patients

**FIGURE 1:**
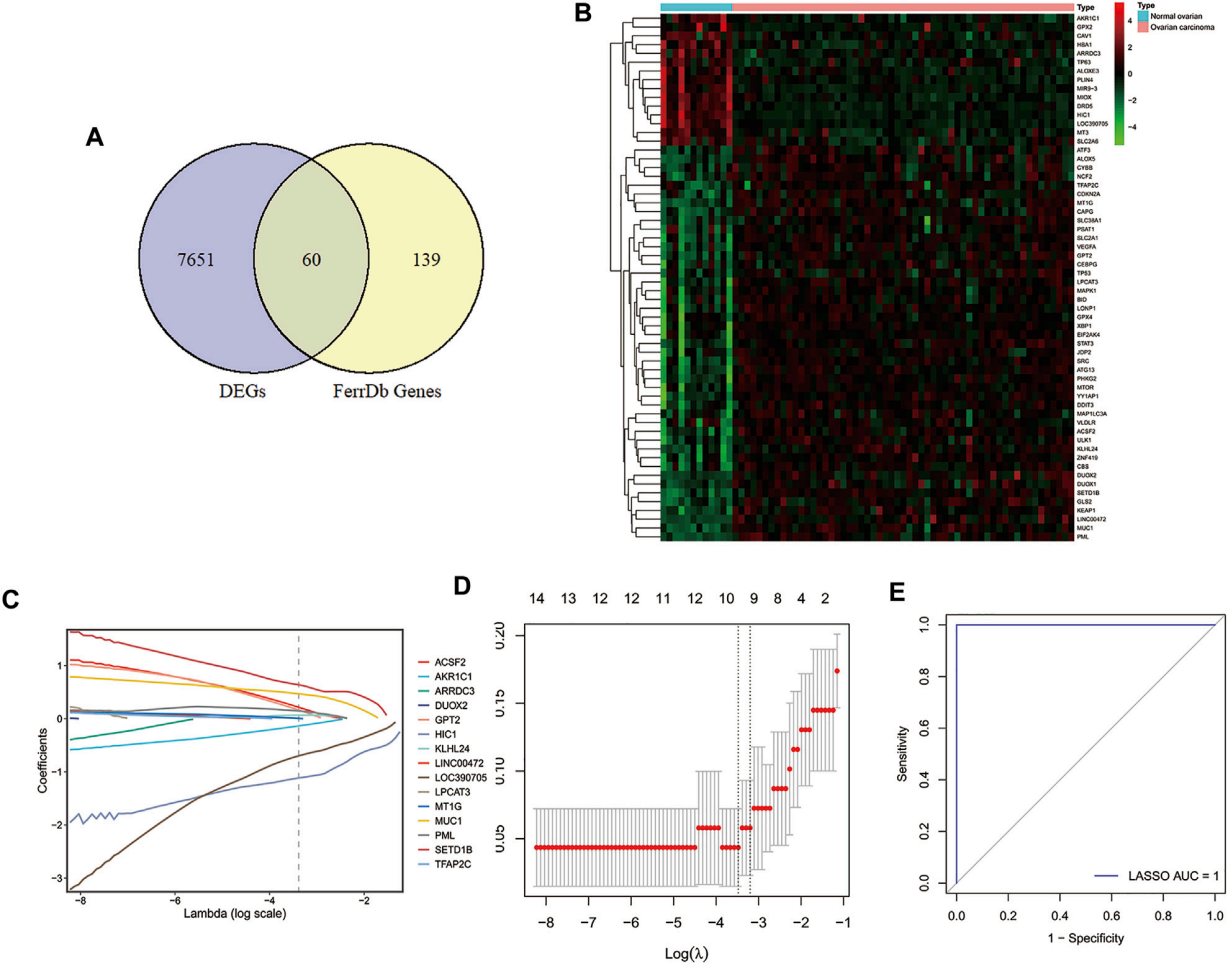
Identification of DE-FRGs and construction of diagnostic signature with FRGs in GEO cohort. **(A)** Venn diagram for identifying DEGs between ovarian carcinoma samples and normal ovaries that belonged to FRGs. **(B)** Heatmap of DE-FRGs in ovarian carcinoma samples compared with normal ovaries. **(C)** Coefficients of the key prognostic FRGs in the LASSO model, each curve represents a gene. **(D)** 10‐fold cross‐validation for tuning parameter selection in the LASSO model. **(E)** ROC curve analysis of the 10-FRG diagnosis signature in GSE66957 dataset. DEGs, differentially expressed genes; DE-FRGs, Differentially Expressed-ferroptosis-related genes; FRGs, ferroptosis-related genes; LASSO, least absolute shrinkage and selection operator; ROC, receiver operating characteristic.

To explore the key genes for the diagnosis of ovarian cancer patients, we performed differential gene analysis between ovarian cancer samples and normal samples based on gene expression profiles from the GSE66957 dataset. A total of 7771 DEGs were identified, of which 4743 genes were up-regulated and 3028 genes were down-regulated in ovarian cancer patients (Supplementary Table S2). Sixty FRGs of them were differentially expressed between tumor tissues and normal tissues (Figure 1A), which were displayed in a heatmap (Figure 1B). Then, we extracted the expression of DE-FRGs to construct the LASSO model based on GSE66957 dataset. As shown in Figures 1C,D, 10 feature genes were screened out, including HIC1, LOC390705, SETD1B, ACSF2, MUC1, KLHL24, PML, MT1G, GPT2, AKR1C1. Among these ten genes, the expression levels of SETD1B, ACSF2, MUC1, KLHL24, PML, MT1G, and GPT2 were higher in tumor tissues than those in normal tissues, while HIC1, AKR1C1, and LOC390705 were lower expressed in tumor tissues. Subsequently, the 10-gene signature was applied to construct a diagnostic model with the LASSO method (Figures 1C,D). The ROC curve analysis was conducted to estimate the validity and precision of the 10-gene signature for the diagnosis of ovarian cancer. Surprisingly, the AUC of these genes signatures was 1.0, demonstrating that the 10-gene signature had the highest diagnostic significance for ovarian cancer (Figure 1E).

### Prognostic Prediction Performance of FRGs in the Patients With Ovarian Cancer

**FIGURE 2:**
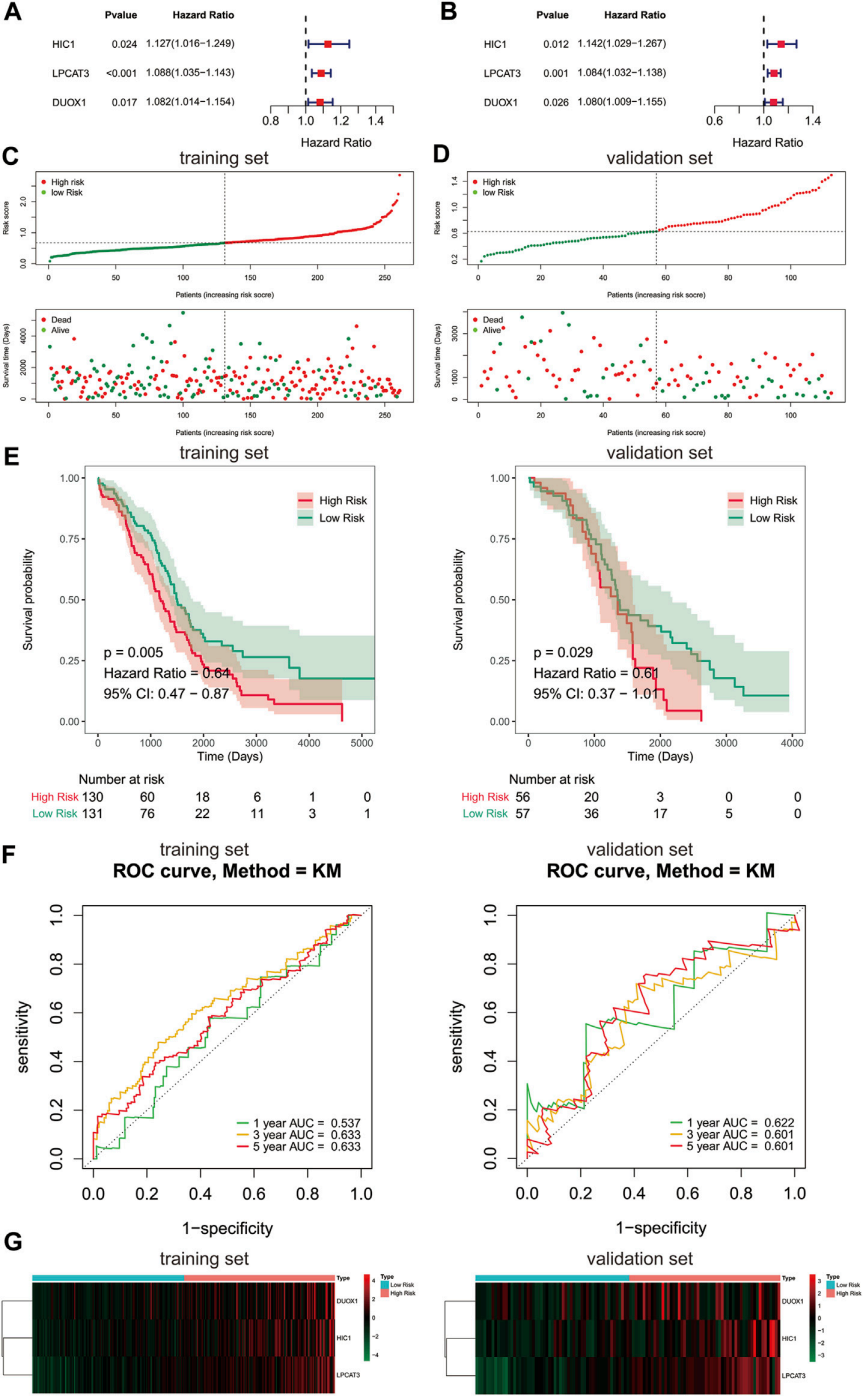
Construction of prognostic gene signature with FRGs in TCGA cohort. **(A, B)** Forest plots showing the results of the univariate and multivariate Cox regression analysis between gene expression and OS in the training set. Distribution of risk score for each patient and survival status of OC patients in the training set **(C)** and validation set **(D)**. **(E)** Kaplan-Meier curves for the OS of patients in the high-risk group and low-risk group in the training set **(left)** and validation set **(right)**. **(F)** The ROC analysis of training set **(left)** and validation set **(right)** for survival prediction by the three-gene signature. **(G)** Heatmap of the gene-expression profiles of the FRGs signature in the training set **(left)** and validation set **(right)**. OS, overall survival; OC, ovarian cancer.

Due to FRGs showed significant diagnostic value in patients with ovarian cancer, we then sought to investigate their relevance to the prognosis of ovarian cancer. We focused on the TCGA database with its clinical information on ovarian cancer. To screen the FRGs that would predict OS in ovarian cancer patients, we performed univariate and multivariate Cox regression analyses on the basis of the 60 FRGs.Finally, a 3-gene signature associated with OS was developed, among which HIC1, LPCAT3, and DUOX1 acted as risk factors (HR > 1) in ovarian cancer (Figures 2A,B). Among them, HICI was found to be a potential diagnostic factor for ovarian cancer.

To further evaluate the prognostic value of the 3-gene signature, a risk score of each sample in the training set and validation set was calculated according to their coefficient and corresponding expression. The ovarian cancer patients of the training set and validation set were stratified into two risk groups based on the median value of risk scores respectively, including the high-risk group (*n* = 130) and the low-risk group (*n* = 131) in the training set, and the high-risk group (*n* = 56) and the low-risk group (*n* = 57) in the validation set. The number of deaths was significantly increased with the increase of risk score in both train and validate cohorts (Figures 2C,D). Kaplan-Meier survival curves demonstrated that the risk scoring system was capable of significantly differentiating the prognostic status of ovarian cancer patients. Patients in the high-risk group showed an obvious worse OS than those in the low-risk group (Figure 2E, *p* < 0.05). Thereafter, the ROC curves showed that the area under the curve (AUC) of 3-gene signature were 0.537, 0.633,0.633 and 0.622, 0.601, 0.601 at 1-,3-, and 5-years in the training and validation set, respectively (Figure 2F). The above evidences suggested that the risk model constructed by the 3-gene signature had tolerable reliability in predicting OS of ovarian cancer. The expressions of 3 signature genes between the two risk groups were displayed in a heatmap (Figure 2G). Surprisingly, the prognostic and diagnostic gene, HIC1 displayed an increased expression level in the high risk group, while it was downregulated in ovarian cancer tissues than normal tissues.

### Independent Prognostic Value of the 3-Gene Signature

**FIGURE 3:**
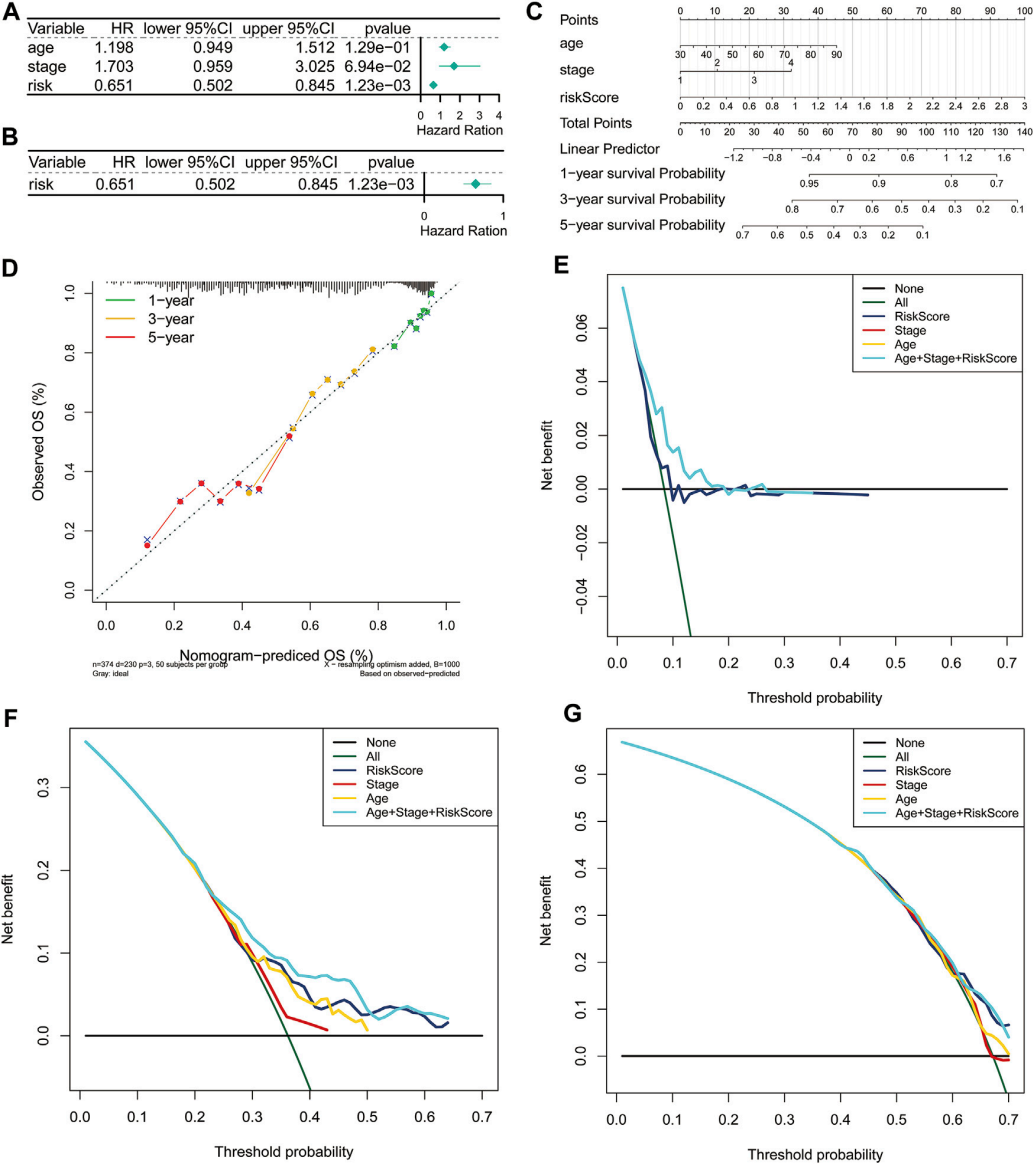
Independent prognostic value of the risk score based on the 3-gene signature**.** Forest plot of univariate **(A)** and multivariate **(B)** Cox proportional hazards regression analysis. **(C)** A nomogram based on risk score and clinical indicators for predicting 1-, 3-, and 5-year OS of ovarian cancer patients in TCGA cohort. **(D)** Calibration plot of nomogram for predicting probabilities of 1-year, 3-year, and 5-year OS of patients. The dotted line indicates actual survival. **(E–G)** Decision curve analysis shows the expected net benefits at 1- **(E)**, 3- **(F)**, and 5-year **(G)** based on the nomogram prediction at different threshold probabilities in the TCGA dataset. None: assume an event will occur in no patients (horizontal solid line); All: assume an event will occur in all patients (green dash line). OS, overall survival.

Furthermore, we carried out univariate and multivariate Cox regression analyses to determine whether the risk score was an independent prognostic predictor for the OS of ovarian cancer patients based on two clinicopathological features (age and stage). In the univariate Cox regression analysis, we found that the risk score was significantly related to the OS of ovarian cancer patients in the TCGA cohort (Figure 3A). More importantly, the risk score was still authenticated to be an independent predictor for ovarian cancer based on the multivariate Cox regression analysis (Figure 3B). The risk score was added to construct a nomogram of 1-, 3- and 5-years survival probability (Figure 3C), and the calibration curves were performed for the nomogram (Figure 3D). Our results indicated that the risk model based on the 3 FRGs could efficiently predict patient survival. DCA also revealed that the risk score benefit was higher than the extreme curve (Figures 3E–G). Meanwhile, risk score combined with clinical features (stage, age) showed a better prediction effect on the prognosis of patients. Taken together, these results revealed that the 3-gene signature showed a significant clinical practical value for patients with ovarian cancer.

### Functional Enrichment Analyses in the TCGA Database

**FIGURE 4:**
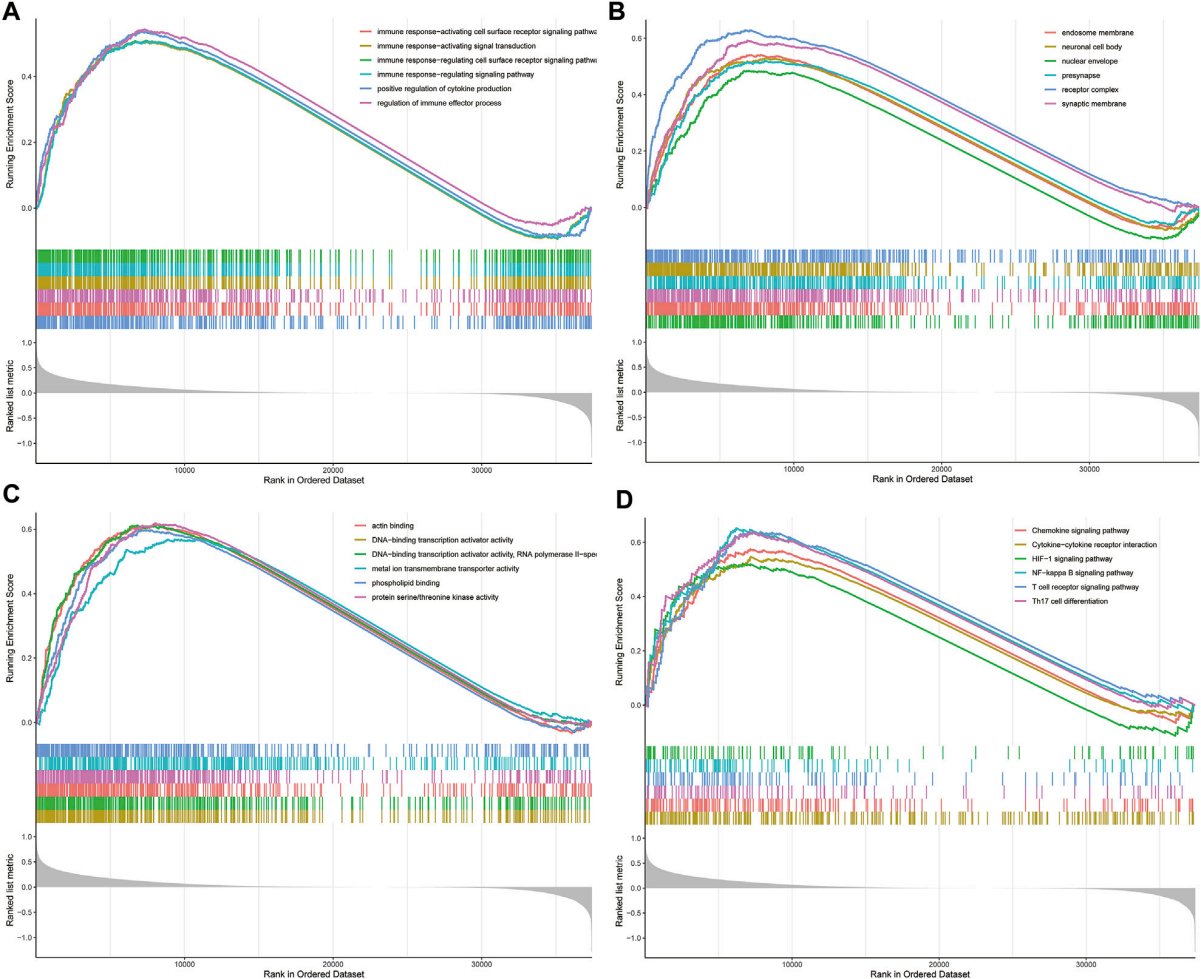
GSEA analysis in TCGA dataset. **(A)** The enriched gene sets in GO-BP category by the DEGs between the two risk groups. **(B)** The enriched gene sets in GO-CC category by the DEGs between the two risk groups. **(C)** Enriched gene sets in GO-MF category by the DEGs between the two risk groups. **(D)** Enrichment plot of the DEGs between the high- and low-risk groups using GSEA-KEGG. Each line representing one particular gene set with unique color, and up-regulated genes located in the left approaching the origin of the coordinates, by contrast the down-regulated lay on the right of *x*-axis. Only gene sets with adjusted *p* < 0.05 were considered significant. And only several leading gene sets were displayed in the plot. GSEA, gene set enrichment analysis; GO, Gene Ontology; BP, biological process; CC, cellular component; MF, molecular function; DEGs, differentially expressed genes; KEGG, Kyoto Encyclopedia of Genes and Genomes.

To elucidate the underlying biological characteristics related to the risk score, GSEA were performed on the basis of the DEGs between the high-risk group and low-risk group in both training and validation sets. The detailed information of GSEA results was exhibited in Supplementary Tables S3–S6. Results of GSEA showed that several immune-related biological processes (BP), such as immune response-activating cell surface receptor signaling pathway, immune response-activating signal transduction, immune response-regulating cell surface receptor signaling pathway, immune response-regulating signaling pathway, positive regulation of cytokine production, regulation of immune effector process were enriched in the high-risk group (Figures 4A–C, *p* < 0.05). In addition, we identified that the DEGs were also involved in many immune-related KEGG pathways, including the T cell receptor signaling pathway, Th17 cell differentiation NF-κB signaling pathway, Chemokine signaling pathway, Cytokine-cytokine receptor interaction, and HIF-1 signaling pathway **(** Figure 4D, *p* < 0.05). Generally, these results implied that ovarian cancer patients in the high- and low-risk groups posses distinct immune characteristics.

### Analysis of the Tumor Microenvironment Between the Two Risk Groups

**FIGURE 5:**
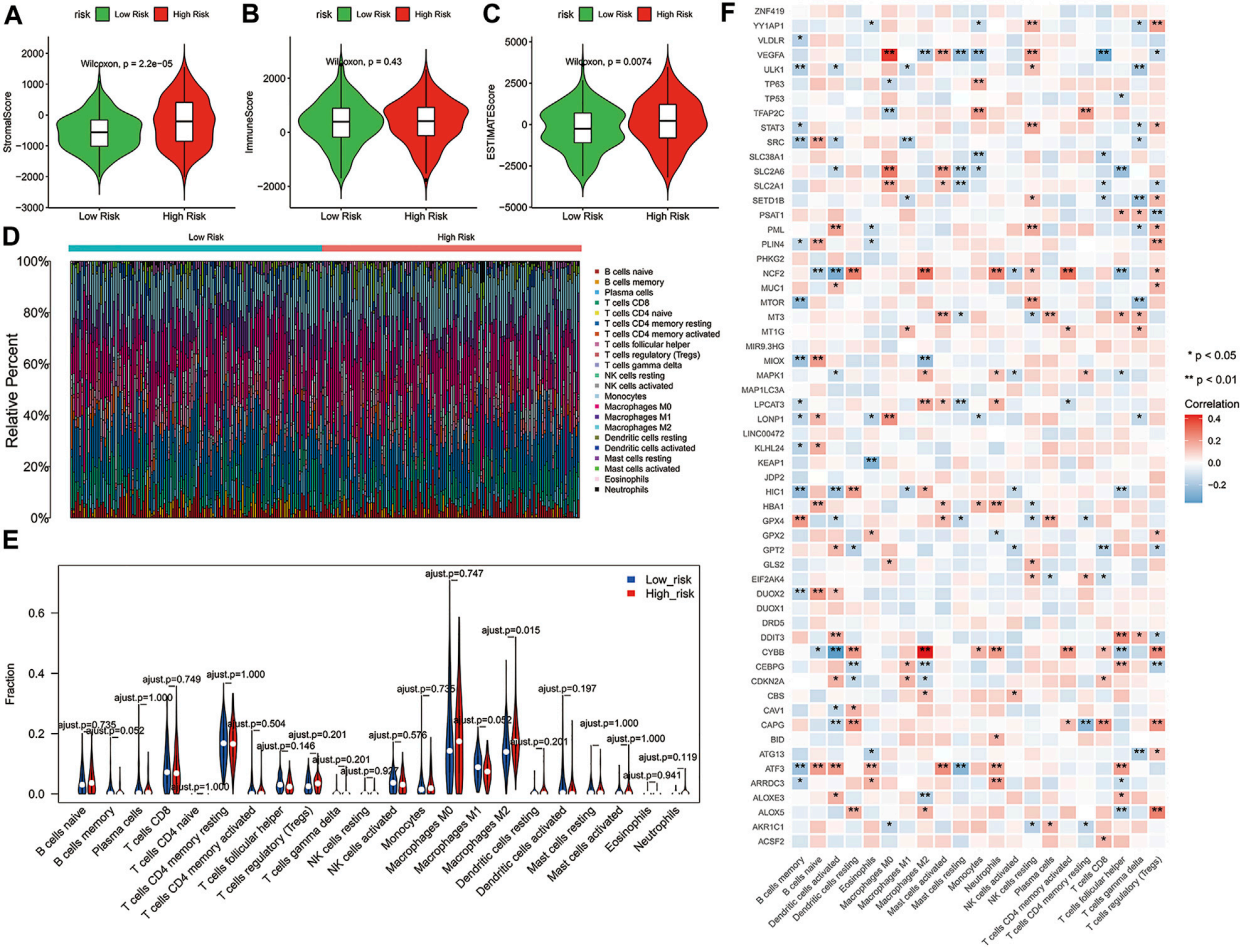
Correlation between the 3-gene signature risk score and immune status. **(A–C)** The differences of Stromal scores, Immune scores and ESTIMATE scores in two risk groups. **(D)** Barplot showed the composition of 22 immune cells in each patient from the high-risk and low-risk groups analyzed by CIBERSORT. **(E)** The Violin plot showed the ratio differentiation of 22 immune cells between OC samples with high or low risk scores, and Wilcoxon rank sum was used for the significance test. **(F)** Heatmap showing the correlation between 22 kinds of immune cells and the DE-FRGs, the asterisk in each tiny box indicating the p value of correlation between two kinds of parameters. The shade of each tiny color box represented corresponding correlation value between two parameters, and Spearman coefficient was used for significance test. ESTIMATE, Estimation of STromal and Immune cells in MAlignant Tumor tissues using Expression data; DE-FRGs, Differentially Expressed-ferroptosis-related genes.

Consistent with our results, emerging evidence had revealed that the immunity displayed an important role in the pathogenesis of ovarian cancer (Menderes et al., 2016). To further explore whether the risk score correlates with the characteristics of TME, we compared the ESTIMATE scores, Stromal scores, and Immune scores of the two risk groups by using the ESTIMATE algorithm. The results indicated that the ESTIMATE scores (*p* = 0.0074) and Stromal scores (*p* = 2.2e-06) had significant differences between the high-risk and low-risk groups, while Immune scores showed no significant differences between the two risk groups (Figures 5A–C). Moreover, the distribution of immune infiltration cells between the two risk groups of ovarian cancer patients was investigated based on the CIBERSORT algorithm, the relative percentage of 22 immune cells in each sample were exhibited in barplot (Figure 5D). The 22 immune cell proportions of ovarian cancer patients in the high- and low-risk groups were displayed the Violin plot, which revealed that the high-risk group showed higher Macrophages M2 infiltration. **(** Figure 5E, *p* < 0.05). In addition, the correlations between the DE-FRGs and 22 tumor-immune cells were analyzed by Spearman analysis (Figure 5F). These results suggested that there was a certain difference in the TME between the high- and low-risk groups, and possibly in the response to immunotherapy.

### Assessment of Response to Immunotherapy and Chemotherapy in Patients With High- and Low-Risk Groups

**FIGURE 6:**
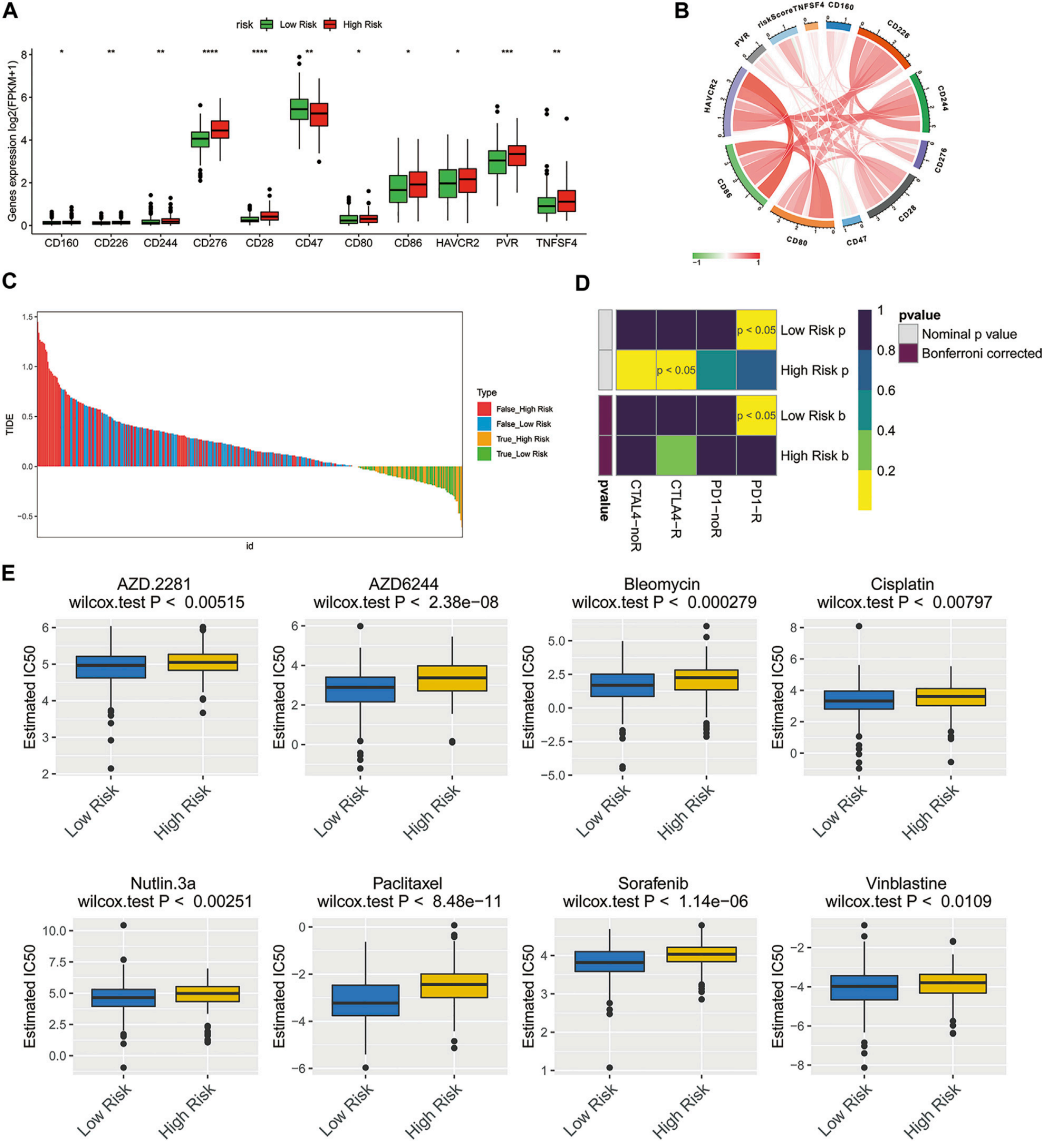
Treatment response prediction of chemotherapy and immunotherapy in the high-risk and low-risk ovarian cancer patients. **(A)** Comparison of the expressions of the immune checkpoint molecules between the low- and high-risk groups. *p < 0.05; **p < 0.01; ***p < 0.001; ****p < 0.0001. **(B)** Circle plot illustrating the association between risk score and main immune checkpoint molecules. **(C)** Immunotherapy response prediction of ovarian cancer patients in the low- and high-risk groups based on TIDE analysis. **(D)** Immunotherapeutic responses to anti-CTLA-4 and anti-PD-1 treatments in high- and low-risk patients. **(E)** IC50 values of 8 typical or potential therapeutic agents for ovarian cancer in the Genomics of Drug Sensitivity in Cancer database for low- and high-risk groups.

Previous studies have shown that immune checkpoint molecules have multiple clinical implications in the course of immunotherapy for tumor patients (De Felice et al., 2015; Odunsi, 2017). Therefore, we determined potential associations between the expressions of key immune checkpoint molecules and two risk groups. Intriguingly, the expressions of all immune checkpoint molecules in the high-risk group were greater than that of another group, except for CD47 **(** Figure 6A, *p* < 0.05), suggesting that high-risk ovarian cancer patients might be more likely to benefit from immunotherapy. The risk score showed correlations with these key immune checkpoint molecules **(** Figure 6B; CD276: Cor = 0.4401, CD28: Cor = 0.3807). Then, the Tumor Immune Dysfunction and Exclusion (TIDE) algorithm and SubMap were applied to predict the clinical response to immune checkpoint blockade (CTLA-4, PD-1, and PD-L1) in different subgroups. TIDE showed that the responses to immune checkpoint blockade were comparable between the high- and low-risk groups **(** Figure 6C **, *p* = 0.9056)**. Interestingly, the ovarian cancer patients of the low-risk group with low expression of immune checkpoint molecules were more likely to be more sensitive to respond to anti-PD-1 therapy **(** Figure 6D **,** Bonferroni corrected *p* < 0.05).

To understand the chemotherapy further comprehensively, the pRRophetic algorithm was used to estimate the response of chemotherapy to the patients of TCGA database. According to the IC50 available in the GDSC database for each TCGA sample, we observed that 59 chemo drugs **(** Supplementary Figure S1 **)** had significant differences in estimated IC50 between the high-risk group and low-risk group, and the patients of the low-risk group were more sensitive to all of these chemotherapies including the most commonly used chemotherapeutic drugs, cisplatin, and paclitaxel, as well as several other potential therapeutic agents **(** Figure 6E **)**.

### Inhibition of HIC1 Improved Drug Sensitivity of Chemotherapy and Anti-PD1 Therapy *via* Inducing Ferroptosis in Ovarian Cancer Cells

**FIGURE 7:**
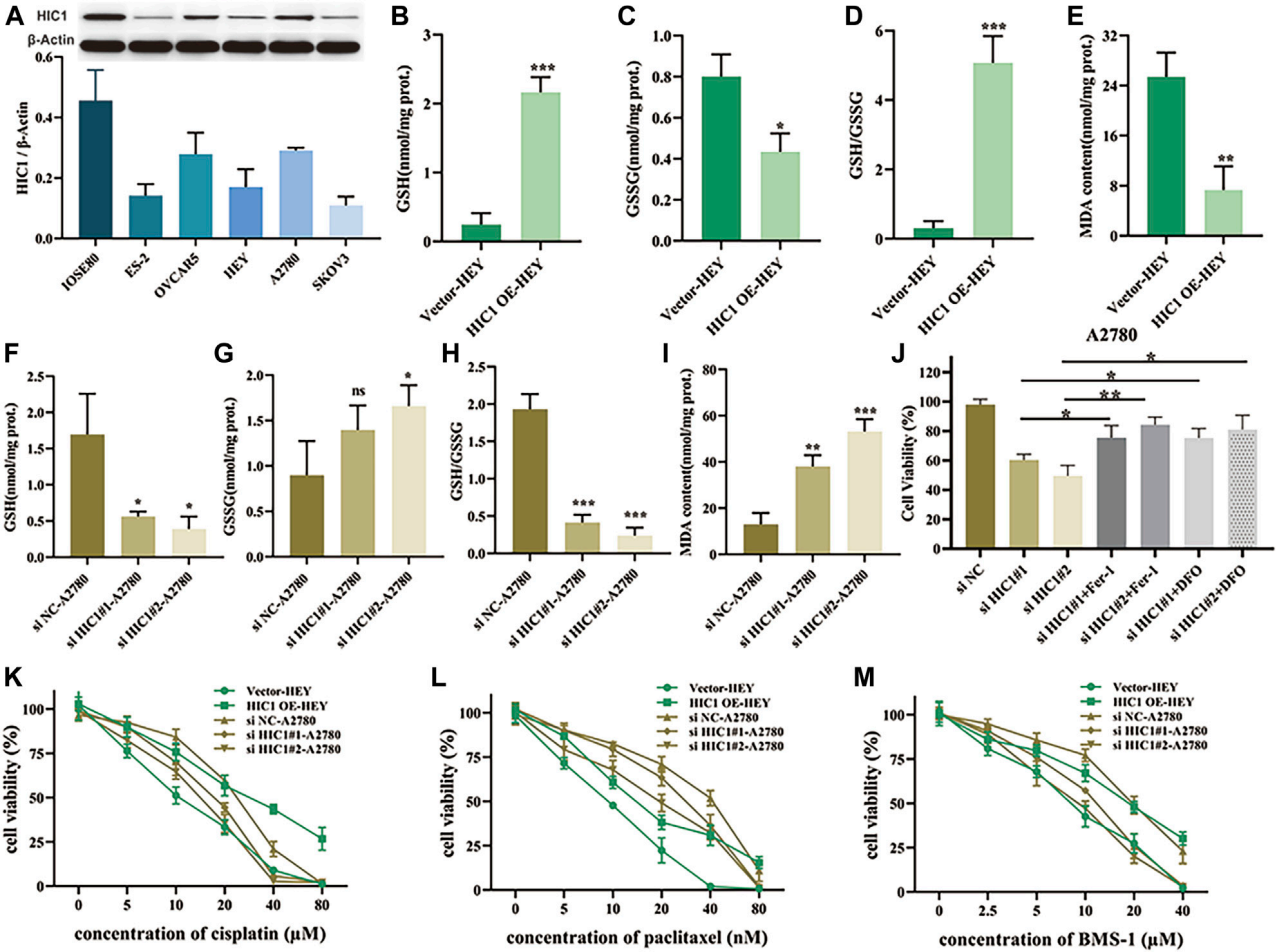
Inhibition of HIC1 improved drug sensitivity of chemotherapy and anti-PD1 therapy *via* inducing ferroptosis in ovarian cancer cells. **(A)** HIC1 expression in five ovarian cancer cell lines (ES-2, OVCAR5, HEY, A2780, and SKOV3) and the human normal ovarian epithelial cell line IOSE80 was detected by Western Blotting. A2780 cells were transfected with negative control siRNA or HIC1 siRNA and then GSH content, GSSG content, the ratio of GSH/GSSG **(B–D)** and MDA content **(E)** were assessed. HEY cells were transfected with empty vector or HIC1 expression plasmid, then GSH content, GSSG content, the ratio of GSH/GSSG **(F–H)** and MDA content **(I)** were detected. **(J)** A2780 cells were treated 2 μM Fer-1 or 20 μM DFO for 24 h after transfected with negative control siRNA or HIC1 siRNAs, then cell viability was evaluated by CCK8 assays. Cell viability was evaluated by CCK8 assays in A2780 cells with or without HIC1 knockdown and HEY cells with or without HIC1 overexpression after treatment with different concentration of cisplatin **(K)**, paclitaxel **(L)**, or BMS-1 **(M)** for 24 h, respectively.

Having found that the FRG, HIC1, exhibited both diagnostic and prognostic value in ovarian cancer. On the basis of this thought-provoking finding, we attempted to explore how HIC1 impacts ferroptosis in ovarian cancer, and whether it relates to treatment responses to chemotherapy and immunotherapy. Our results showed that the expression of HIC1 was lower in ovarian cancer cell lines (ES-2, OVCAR5, HEY, A2780, and SKOV3) as compared to that in the normal ovarian cell line (IOSE80). While, A2780, OVCAR5 showed higher expression of HIC1 than HEY, ES-2, and SKOV3 cells **(** Figure 7A **)**. Then, HIC1 high expressed cell-A2780, and HIC1 low expressed cell-HEY, were used to perform the following experiments. We genetically downregulated or upregulated HIC1 expression in A2780 and HEY cells. The efficiency of HIC1 knockdown and overexpression was verified **(** Supplementary Figure S2 **)**. As is shown in Figure 7, HIC1 knockdown in A2780 cells significantly reduced GSH/GSSG ratio **(** Figures 7B–D **)** and improved MDA content **(** Figure 7E **)**. On the contrary, the opposite results were observed for HIC1 overexpression in HEY cells **(** Figures 7F–I **)**. In addition, we found that cell death induced by HIC1 knockdown could be reversed by ferroptosis inhibitors, ferrostatin-1 and DFO (Figure 7J). The above results suggests that inhibition of HIC1 induced ferroptosis. Consequently, we tested whether HIC1 affects the drug sensitivity of conventional chemotherapy and anti-PD1 agents in ovarian cancer cells. The results showed that inhibition of HIC1 made A2780 cells were more sensitive to cisplatin (Figure 7K), paclitaxel (Figure 7L), and BMS-1 (Figure 7M). While overexpression of HIC1 in HEY cells resulted in drug resistance to those agents.

## Discussion

Aberrant regulation of cell death programs plays a pivotal role in tumorigenesis and tumor development. As we know, resistance to apoptosis is a hallmark of cancer (Frank et al., 2019; Koren and Fuchs, 2021). Thus, over the past decades, scholars committed to identifying and clarifying the regulatory mechanism of nonapoptotic death forms to improve the diagnosis and treatment of cancer. As a newly identified type of regulated cell death (RCD), ferroptosis attracted increasing attention for its critical role in numerous diseases including cancer. Ovarian cancer is a highly heterogeneous tumor with substantial somatic mutations, accounting for the highest mortality rate among all gynecological malignancies (Yang et al., 2017). Our previous work indicated that ferroptosis inducer erastin synergistically induced ovarian cancer cell death with cisplatin which manifested that targeting ferroptosis in ovarian caner offers therapeutic perspectives (Cheng et al., 2021). In this regard, we speculated that FRGs may be involved in the oncogenesis and development of OC. In the present study, we systemically investigated the clinical relations, TME features, and treatment response of ferroptosis patterns in OC.

Based on the data from GEO and TCGA databases, we constructed a novel FRGs signature for the diagnosis and prognosis of OC, respectively. Here, a 10-FRG diagnostic signature was constructed, including HIC1, LOC390705, SETD1B, ACSF2, MUC1, KLHL24, PML, MT1G, GPT2, and AKR1C1. According to previous reports, LOC390705 was found to be downregulated, while KLHL24 and GPT2 were upregulated in erastin treated in HT-1080 cells and SETD1B was enriched in GPX4 inhibitor ML162-resistant cells (Dixon et al., 2014; Dixon et al., 2015). Besides, HIC1 (Zhang et al., 2019) MUC1 (Hasegawa et al., 2016), PML (Saint-Germain et al., 2017), MT1G (Sun et al., 2016), and AKR1C1 (Dixon et al., 2014; Gagliardi et al., 2019) were proved to act as suppressors and ACSF2 as driver of ferroptosis (Dixon et al., 2012). Our diagnostic models based on these 10 genes showed high sensitivity and specificity, which may contribute to the early diagnosis of ovarian cancer. More importantly, we identified a 3-FRG (HIC1, LPCAT3, DUOX1) signature to predict the prognosis of OC patients. Meanwhile, these 3 FRGs were applied to construct a prognostic risk model, of which predictive capacity was proved to be reliable. Ovarian cancer patients were stratified into high-risk and low-risk subgroups according to the risk score calculated by the signature. Patients in the high-risk group showed a shorter OS and a worse prognosis. Above all, we had developed robust FRGs signatures for diagnosis as well as predicting the outcome of OC patients.

Unexpectedly, the ferroptosis-related gene, HIC1, was proved to possess both diagnostic and prognostic value for OC patients in our work. Our results showed that HIC1 was down-regulated in ovarian cancer patients, but had increased expression level in the high-risk group patients with poorer outcome. We speculate the opposite action of HIC1 in affecting tumor initiation and patient outcome may due to the context-specific manner by HIC1 in regulating biological process through different mechanisms. Recently, a similar pattern of results was obtained in gastric cancer (GC) that INPP4B may play dual roles as an oncogene and tumour suppressor gene in different conditions. INPP4B was found to be expressed lower in GC tissues compared with nontumour tissues. Contradictorily, GC patients with high expression of INPP4B had a better prognosis in the well differentiated tissue grade and early clinical stage but had a poor prognosis in the worse tissue grade and advanced clinical stage (Wu et al., 2021). HIC1 (Hypermethylated in cancer 1) is known as a tumor-suppressor gene, which is implicated in many canonical processes of cancer such as cell growth, cell survival, cell migration, and motility. Its expression was commonly silenced or down-regulated due to the CpG island hypermethylation in various malignancies (Rood and Leprince, 2013)including colon cancer (Janeckova et al., 2015), breast cancer (Wang et al., 2018), and prostate cancer (Hao et al., 2017). A prior study showed that the combined detection of HIC1 with another tumor suppressor gene, HOXA9, possessed great potential for the recognition of ovarian cancer (Singh et al., 2020). Additionally, HIC1 expression was found to be silenced only in triple-negative breast cancer (TNBC) compared with other molecular subtypes of breast cancer, HIC1 contributed to reduced cell migration, invasion and metastasis in triple-negative breast cancer (TNBC) cells (Cheng et al., 2014). To elucidate the interaction of HIC1 and ferroptosis in clinical prognostic significance for ovarian cancer, we carried out *in vitro* experiments. On the one hand, we confirmed that expression of HIC1 was reduced in ovarian cancer cells compared to normal ovarian cells. On the other hand, our results illustrated that knockdown of HIC1 improved drug sensitive of chemotherapy and immunotherapy by inducing ferroptosis, which suggest a potential role for HIC1 in the treatment of OC through meditating ferroptosis. Meanwhile, we found that compared to empty vector, overexpression of HIC1 reduced drug sensitivity of cisplatin, paclitaxel and BMS-1. We speculate that this phenomenon was mainly due to the three agents may induce ferroptosis in varying extents. However, a previous research considered HIC1 was a ferroptosis driver gene for HIC1 stimulated ferroptosis through regulating GSH synthesis, and subsequently leading to inhibition of tumor growth in liver cancer (Zhang et al., 2019). The inconsistent results reflected that regulation of ferroptosis by HIC1 appeared to be cancer-type dependent.

Notably, GSEA of the low- and high-risk group constructed based on the 3 differentially expressed FRGs revealed that immune-related processes and pathways achieved high enrichment scores in the high-risk groups. Moreover, TME (tumor microenvironment) analysis of OC patients indicated that ESTIMATE score which respresents tumor purity was significantly higher in the high-risk group. As is known to all, the TME is of major importance in tumor immunity, which is considered to be critical for tumor cell fate determination. Tumor-associated macrophages (TAMs) were important components of the tumor immune microenvironment, including M1-and M2-polarized macrophages, which function as anti-tumoral and pro-tumoral, respectively (Cheng et al., 2018). A recent study has shown that H_2_O_2_ induced autophagy-dependent ferroptotic cell death drives tumor-associated macrophage to polarize into M2 phenotype *via* improving extracellular KRASG12D release, which promotes human pancreatic ductal adenocarcinoma progression (Dai et al., 2020). In line with this, current research exhibited that OC patients in the high-risk group had increased infiltration level of M2 macrophages as compared to that in the low-risk group.

To date, therapeutic options remain limited in OC with high rates of recurrence and chemoresistance. Immunotherapy represents one of the next frontiers in cancer. Previously published studies have shown that ferroptosis was closely related to tumor immunotherapy (Lang et al., 2019; Wang et al., 2019). Immune checkpoint blockade, as one of the most impactful classes of immunotherapy, has also drawn increasing attention from researchers engaged in ferroptosis (Stockwell and Jiang, 2019). But until now, only a limited number of patients exhibit a durable clinical benefit from immune checkpoint blockade. PD-1/PD-L1 and CTLA-4 are the best characterized and most clinically studied immune checkpoints so far (Chae et al., 2018; Martí i Líndez et al., 2019). Interestingly, our investigation of response to treatment with checkpoint-blocking antibodies targeting CTLA-4 and PD-1/PD-L1 in the two subgroups illustrated that patients in the low-risk group with low expression of immune checkpoint molecules were more likely to be sensitive to anti-PD1 therapy. Indeed, in clinical practice, quite a few PD-L1 positive patients respond poorly to the PD-1/PD-L1 treatment, while some patients with negative PD-L1 have a surprising response to treatment (Li et al., 2021). We speculate that the mechanism of action of immune checkpoint inhibitors is complicated, but not simply targeting immune checkpoint.

Among the 3 gene signatures, HIC1, LPCAT3, and DUOX1 in our prognostic model, HIC1 is the most investigated gene correlated with cellular immune function. A previous study identified HIC1 as a regulator of intestinal immune responses under homeostatic and inflammatory conditions and hinted a critical role for HIC1 in the pathogenicity of T cells (Burrows et al., 2017). In line with this, another study revealed that HIC1 participated in human iTreg cell differentiation *via* binding to the promoters of transcription factors required for Th1/2/17 cell development and repressed their transcription, which suggested that HIC1 may play an important role in intestinal homeostasis by maintaining Treg cell suppressive ability to sustain tolerance to innocuous antigens (Ubaid Ullah et al., 2018). Despite the role of HIC1 in cellular immunity, whether the other two genes in our risk model regulates immune function remians unknown. Coincidentally, the most recent studies discovered that the anti-tumor function of the immune system may associated with ferroptosis. In a recent research aimed at exploring whether ferroptotic cancer cells are immunogenic demonstrated that early stage ferroptotic cancer cells induced by RSL3 were efficiently engulfed by bone marrow-derived dendritic cells (BMDCs) and were able to promote BMDCs maturation and activation. Moreover, early ferroptotic cancer cells in a tumor prophylactic vaccination model showed effective vaccination activity in immune-competent mice. This study provides evidence to support that ferroptotic cancer cells in the early death stages can acts as effective inducers of an adaptive immune response (Efimova et al., 2020). Another study revealed that CD8^+^ T cells released interferon-gamma (IFNγ) promoted ferroptosis-specific lipid peroxidation in tumor cells *via* downregulating the expression of SLC3A2 and SLC7A11, and in turn, increased ferroptosis contributes to the anti-tumor efficacy of immunotherapy. The study also proved that ferroptosis inducer in conjunction with checkpoint blockade synergistically enhanced T cell-mediated anti-tumor immunity and ferroptosis in the preclinical model (Wang et al., 2019). Findings discussed above along with our results raised the question of whether immune cells provide a linkage in ferroptosis regulated by the three prognostic genes, HIC1, LPCAT3, and DUOX1 in ovarian cancer, this issue deserves further investigation.

In summary, we comprehensively elucidated the role of novel FRGs signature in diagnosis and prognosis in ovarian cancer for the first time. Specifically, a 10-gene signature (HIC1, ACSF2, MUC1, etc.) was developed for the diagnosis of ovarian cancer with high sensitivity using LASSO regression. Meanwhile, we constructed a novel prognostic signature consisting of three FRGs (HIC1, LPCAT3, DUOX1), The three FRG-based risk score model was capable of distinguishing ovarian cancer patients with significantly different outcomes, and the risk score was the independent prognostic factor. Function analyses highlighted the tight correlation between the risk score and tumor immunity in ovarian cancer. Besides, the diagnostic and prognostic gene, HIC1, may represent a potential therapeutic target for ovarian cancer. Our work provides new insight into early detection, prognostic prediction, and guiding individualized treatment of patients with ovarian cancer.

## Data Availability

The datasets presented in this study can be found in online repositories. The names of the repository/repositories and accession number(s) can be found in the article/Supplementary Material.

## Supplementary Material

Supplementary Figure S1 Drug efficiency of therapeutic agents with the significant difference in the Genomics of Drug Sensitivity in Cancer database for low- and high- risk groups.

Supplementary Figure S2 Knockdown and overexpression efficiency of HICI.

Supplementary Table S1 A detailed list of DE-FRGs based on intersection of the DEGs acquired from the GSE66957 dataset with FRGs.

Supplementary Table S2 A detailed list of DEGs in the GSE66957 dataset.

Supplementary Table S3 The detailed results of GSEA(GO_BP).

Supplementary Table S4 The detailed results of GSEA(GO_CC).

Supplementary Table S5 The detailed results of GSEA(GO_MF).

Supplementary Table S6 The detailed results of GSEA(KEGG pathways).
