# Supplementary figures and images for: Integrated Analysis of Ferroptosis-Related Biomarker Signatures to Improve the Diagnosis and Prognosis Prediction of Ovarian Cancer
Source: Front Cell Dev Biol. 2022 Jan 5;9:807862. doi: 10.3389/fcell.2021.807862 (PMC8766510; doi:10.3389/fcell.2021.807862)

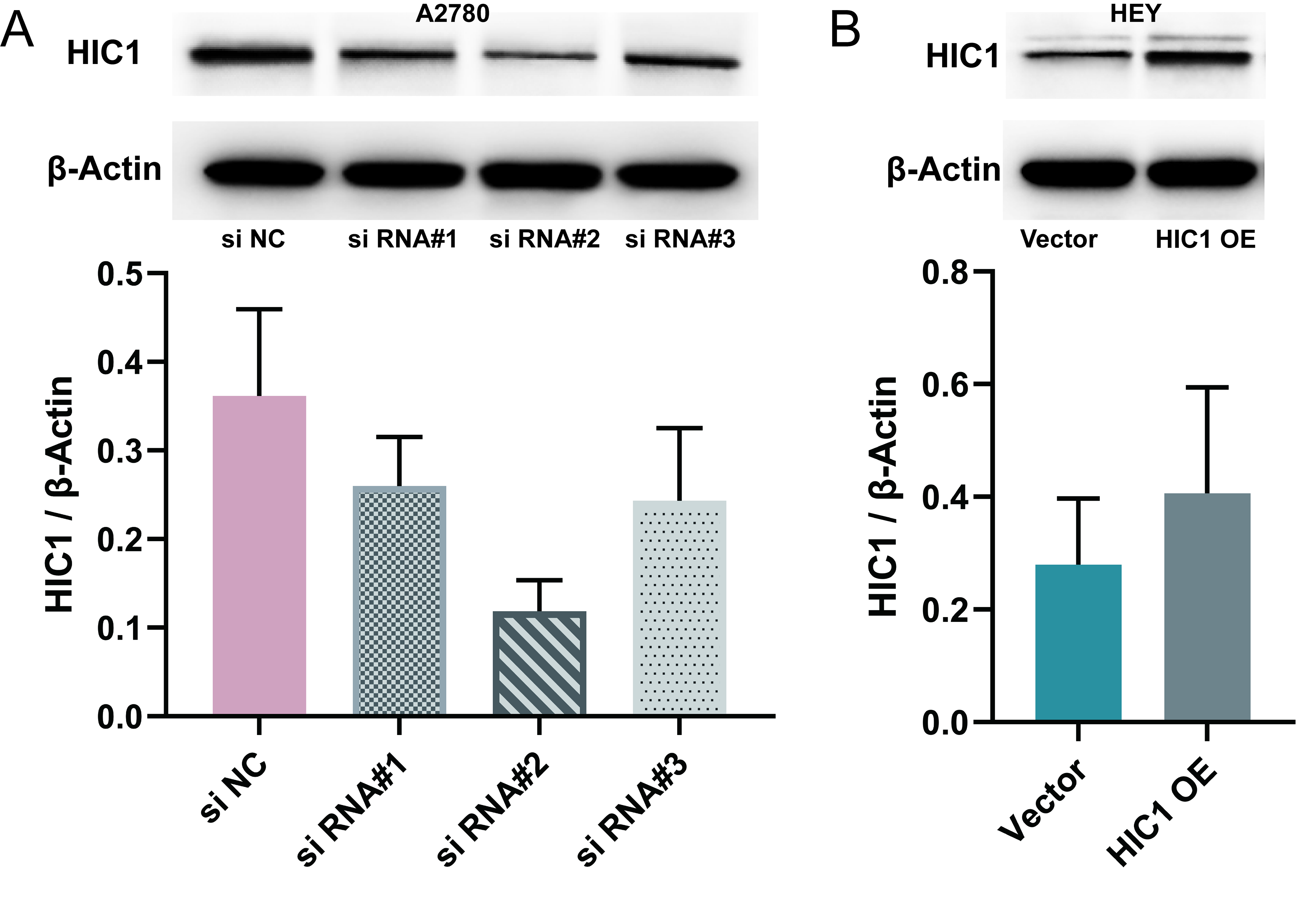

Supplement: Supplementary file 4 [file Image2.TIF]

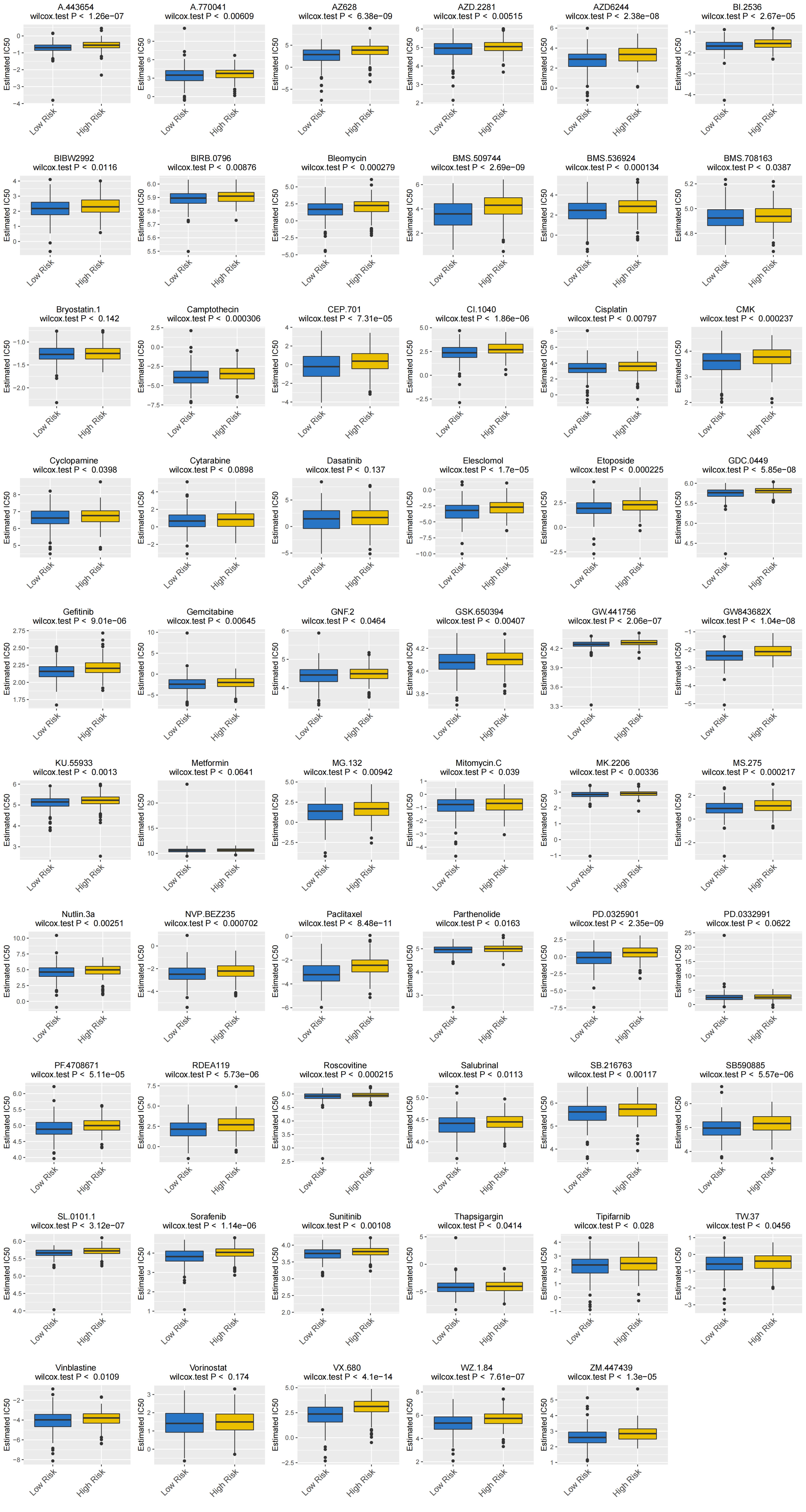

Supplement: Supplementary file 6 [file Image1.TIF]
